# Sodium Hydrosulfide Induces Resistance Against *Penicillium expansum* in Apples by Regulating Hydrogen Peroxide and Nitric Oxide Activation of Phenylpropanoid Metabolism

**DOI:** 10.3389/fmicb.2021.720372

**Published:** 2021-09-01

**Authors:** Huiwen Deng, Bin Wang, Yongxiang Liu, Li Ma, Yuanyuan Zong, Dov Prusky, Yang Bi

**Affiliations:** ^1^College of Horticulture, Gansu Agricultural University, Lanzhou, China; ^2^College of Food Science and Engineering, Gansu Agricultural University, Lanzhou, China; ^3^Department of Postharvest Science of Fresh Produce, Agricultural Research Organization (ARO), Volcani Center, Rishon LeZion, Israel

**Keywords:** apple fruit, NaHS, *Penicillium expansum*, induced resistance, signaling molecule, phenylpropanoid metabolism

## Abstract

As a multifunctional signaling molecule, hydrogen sulfide (H_2_S) has been reported to induce plant responses to a variety of abiotic stresses. However, there are no reports on H_2_S treatment inducing resistance in apples against *Penicillium expansum*, a biotic factor, and its possible mechanism of action. In this study, fumigating apples with 5 mM sodium hydrosulfide (NaHS), the exogenous donor of H_2_S, for 12 h reduced the diameter of lesions in fruit colonized by *P. expansum*. NaHS treatment markedly promoted the synthesis of endogenous H_2_S, hydrogen peroxide (H_2_O_2_), and nitrogen oxide (NO). *In vivo* NaHS treatment enhanced the activities of phenylalanine ammonia-lyase, cinnamate 4-hydroxylase, *p*-coumarate:coenzyme A ligase isoenzymes, caffeoyl-CoA-O-methyltransferase, caffeic acid-O-methyltransferase, ferulic acid-5-hydroxylase, cinnamyl-CoA reductase, and cinnamyl-alcohol dehydrogenase. The treatment also facilitated the production of specific phenolic acids, such as cinnamic acid, *p*-coumaric acid, caffeic acid, ferulic acid, and sinapic acid; total phenolic compounds; *p-*coumaryl alcohol; coniferyl alcohol; sinapyl alcohol; and lignin. NaHS treatment induced resistance against *P. expansum* in apples through H_2_O_2_- and NO-mediated activation of phenylpropanoid metabolism.

## Introduction

*Penicillium expansum* is a critical pathogen that can cause blue mold in various temperate fruits (Errampalli, 2014), resulting not only in fruit rot but also in the accumulation of patulin in the fruit, which presents a potential safety hazard (Yu et al., 2020). Although *P. expansum* can be effectively controlled by the fungicides pyrimethanil and thiabendazole (Ghosh et al., 2006; Sugar and Basile, 2008), pesticide residues remain on the fruit after storage and pose a risk to human health and the pathogens can possible develop resistance to them. Moreover, there may be registration difficulties (Romanazzi et al., 2016). These reasons make it necessary to exploit new alternative strategies for preventing postharvest diseases.

Induced resistance is a novel, valid approach that has been applied to control postharvest diseases of fruit and vegetables by triggering natural resistance using elicitors and increasing host resistance against pathogens (Bi et al., 2020). Hydrogen sulfide (H_2_S) is a signaling molecule that is extensively found in plants and has a protective function in stress responses (Yamasaki and Cohen, 2016). Studies have shown that exogenous H_2_S and NaHS treatments potentially enhance plant resistance against various biological or abiotic stresses (Wang, 2012; Guo et al., 2016). NaHS treatment significantly inhibits the decay of fresh-cut pears caused by *P. expansum* and *Aspergillus niger* (Hu K. D. et al., 2014); alleviates fresh-cut sweet potato rot induced by *Geotrichum candidum*, *Mucor rouxianus*, and *Rhizopus nigricans* (Tang et al., 2014); and sustains a lower rot index in strawberry fruit (Hu et al., 2012). Other studies have indicated that NaHS treatment improves the activity of L-cysteine desulfhydrase (LCD) in *Arabidopsis thaliana* and promotes the generation of endogenous H_2_S, which regulates the downstream synthesis of salicylic acid, a signal molecule (Shi et al., 2015); reduces the activity of phenylalanine ammonia-lyase; enhances the activity of ascorbate peroxidase, catalase, and peroxidase; and eliminates excessive reactive oxygen species, all of which preserves the stability of the cell membrane (Hu K. D. et al., 2014). Previous molecular analyses revealed that the delayed postharvest senescence of apples caused by H_2_S was linked to the suppression of the genes involved in ethylene biosynthesis (*MdACS1*, *MdACS3*, *MdACO1*, and *MdACO2*) and signal transduction (*MdETR1*, *MdERS1*, *MdERS2*, *MdERF3*, *MdERF4*, and *MdERF5*); these results supported the proposed counteractive role of H_2_S in ethylene biosynthesis and signaling (Ziogas et al., 2018). However, H_2_S treatment in the present work induces the resistance of apples to *P. expansum*, and its mechanism of action has not been investigated yet.

The objectives of this study were to evaluate the effect of NaHS treatment on the colonization pattern of *P. expansum* in treated apples and analyze the possible mechanism of action by evaluating the levels of H_2_S, hydrogen peroxide (H_2_O_2_), and nitrogen oxide (NO) and the activation of the enzymatic products of phenylpropanoid metabolism.

## Materials and Methods

### Materials

Apples (*Malus domestica* Borkh cv. Delicious) were picked from a state-owned orchard in the Tiaoshan farm in Jingtai County, Gansu Province, China. The fruits selected were of similar size and without any defects. A total of 360 fruits were randomly divided into two groups. Each group included three replicates of 60 fruits. They were placed in transport boxes (45 apples per box), delivered to the laboratory on the day of harvesting, and maintained in cold storage (4°C ± 2°C; RH, 55–65%). *P. expansum* T01 was donated by Professor Tian Shipping of the Institute of Botany, Chinese Academy of Sciences, and preserved on potato dextrose agar at 25°C. NaHS (AR 68%) was bought from Shanghai Macklin Biochemical Technology Co., Ltd. HPLC-grade standards were purchased from Nanjing Yuanzhi Biotechnology Co., Ltd.

### NaHS Treatment

Following previous screening study protocols (Li D. et al., 2016), a 5-mM solution of NaHS was prepared using distilled water. Accordingly, 50 ml of 5 mM NaHS solution was placed in a 90-mm-diameter container that was then placed in a 13-L desiccator. NaHS treatment was conducted following the method of Li D. et al. (2016) with appropriate modifications. The fruits were placed in ambient temperature (22°C ± 2°C; RH, 55–60%) for approximately 24 h and washed twice to remove dirt, after which they were dipped in 0.1% (v/v) sodium hypochlorite for 2 min. The fruits were washed again with tap water and dried at ambient temperature (22°C ± 2°C; RH, 55–60%) for 2 h. Then, the fruits were fumigated with NaHS solutions in a desiccator for 12 h with distilled water acting as control. All treated fruits were placed in a carton and stored at ambient temperature (22°C ± 2°C; RH, 55–60%). Overall, 48 fruits were randomly selected for each treatment with three replicates.

### Inoculation

The spore suspension was prepared according to the method of Tahir et al. (2015). Herein, 5 ml of sterile water was added to a 5-day-old culture plate of *P. expansum* and the spores were scraped off and filtered through four layers of germ-free gauze to obtain a suspension of 1 × 10^6^ spores/ml.

Inoculation and measurement of lesion diameters were conducted as per the method of Li S. E. et al. (2019). All fruits were disinfected using 75% ethanol, and four uniform piercings were made using a sterilized nail (3 mm × 3 mm). After 24 h of treatment, 20 μl of the prepared spore suspension was inoculated into the wound. The fruits were dried and then placed at ambient temperature (22°C ± 2°C; RH, 55–60%). Lesion diameters were measured at 3, 4, 5, 6, and 7 days after inoculation. The longest and shortest diameters of the lesion area were determined by the crossover method and the average was taken as the lesion diameter. Three replicates of 12 randomly selected fruits per treatment method were examined.

### Sampling

Sampling was performed as per the method of Ge et al. (2017). After 0, 2, 4, 6, 8, and 10 days of treatment, the test and control fruits were peeled, and using a stainless steel knife, approximately 2.0 g of fresh tissue was collected from 2 to 3 mm below the epidermis at the equatorial part for each fruit. The samples were then pulverized into powder after adding liquid nitrogen and stored at −80°C in 50 ml centrifuge tubes.

### Endogenous H_2_S, NO, and H_2_O_2_ Content Assays

Endogenous H_2_S content was determined following the method of Sekiya et al. (1982) with appropriate modifications. Frozen tissue powder (1 g) was homogenized in 1 ml of 50 mM PBS (pH = 6.8) extracting solution, including 100 mM EDTA-Na_2_ and 200 mM ascorbic acid. The homogenate (1 ml) was placed in 0.5 ml of 1 M HCl to release H_2_S, which was absorbed by 0.5 ml of 1% (w/v) zinc acetate. After allowing the reaction to occur for 30 min, 0.3 ml of 5 mM dimethyl-p-phenylenediamine dihydrochloride dissolved in 3.5 mM H_2_SO_4_ was added, followed by 0.3 ml of 50 mM NH_4_Fe(SO_4_)_2_ in 100 mM H_2_SO_4_. The absorbance was measured at 667 nm after incubating for 15 min at ambient temperature (22°C ± 2°C). Results are presented on a fresh weight basis, and the endogenous H_2_S content was represented as mol kg^–1^.

Nitrogen oxide content was determined using a kit from the Suzhou Keming Biotechnology Co., Ltd., following the protocols of the manufacturer. Frozen tissue powder (0.5 g) was placed in 1 ml of extracting solution, and then centrifuged at 10,000 × *g* for 15 min at 4°C. The reaction system included 100 μl of the supernatant and 50 μl of reagents 1 and 2, respectively. After incubating at ambient temperature (22°C ± 2°C; RH, 55–60%) for 15 min, the absorbance was measured at 550 nm, with extracting solution as a control. Results are expressed on a fresh weight basis, and the NO content was expressed as mmol kg^–1^.

H_2_O_2_ content was measured using the method of Prochazkova et al. (2001) with appropriate modifications. Frozen tissue powder (1 g) was homogenized in 1 ml of cooled acetone and centrifuged at 10,000 × *g* for 40 min at 4°C. The reaction system included 1 ml of the supernatant, 100 μl of 20% titanium tetrachloride solution (v/v), and 200 μl of ammonia solution. It was homogenized for 5 min and centrifuged at 10,000 × *g* for 15 min at 4°C. The sediment was washed four times with cooled acetone and dissolved in 1.5 ml of 1 mM H_2_SO_4_. Absorbance was measured at 410 nm. Results are represented on a fresh weight basis, and the H_2_O_2_ content was represented as mmol kg^–1^.

### Measurement of Enzyme Activity

The activity of phenylalanine ammonia-lyase (PAL) was assayed following the method of Koukol and Conne (1961) with appropriate modifications. Frozen tissue powder (2 g) was homogenized in 2 ml of 0.1 M sodium borate buffer (pH = 8.8), including 5 mM β-mercaptoethanol (BME), 2 mM EDTA, and 4% polyvinylpyrrolidone (PVP) for PAL. The homogenates were centrifuged at 10,000 × *g* for 40 min at 4°C and the supernatant was used as a crude enzyme extract. The reaction system included 3 ml of buffer solution, 500 μl of L-phenylalanine, and 500 μl of crude enzyme extract. These were incubated for 1 h at 40°C, and the reaction was terminated by adding 200 μl of 6 M HCl. Absorbance was measured at 290 nm and PAL activity was represented as U mg^–1^ protein.

The activities of cinnamate 4-hydroxylase (C4H) and *p*-coumarate:coenzyme A ligase isoenzymes (4CL) were determined using the method of Voo et al. (1995) with appropriate modifications. Frozen tissue powder (2 g) was placed in 2 ml of extracting solution containing 5 mM BME, 4 mM MgCl_2_, 50 mM Tris–HCl, 10% glycerinum, 10 μM Leupeptin, 1 mM PMSF, 5 mM ascorbic acid, and 0.15% PVP to crude extracts of C4H and 4CL enzymes. The C4H reaction system included 2 ml of 0.05 M Tris–HCl (pH = 8.9) containing 2 μM *trans-*cinnamic acid, 2 μM NADPNa_2_, 5 μM D-glucose 6-phosphate disodium salt, and 800 μl of crude enzyme extract. The reaction system of 4CL included 150 μl of 75 mM MgCl_2_, 150 μl of 1 μM CoA, 150 μl of 80 M ATP, 150 μl of 20 mM *p-*coumaric acid, and 500 μl of crude enzymes extract. The absorbance of C4H and 4CL were measured at 340 and 333 nm, respectively, and the activities of C4H and 4CL were expressed as U mg^–1^ protein.

The activities of caffeoyl-CoA-O-methyltransferase (CCoAOMT), caffeic acid-O-methyltransferase (COMT), ferulic acid-5-hydroxylase (F5H), and cinnamyl-CoA reductase (CCR) were measured using the enzyme-linked immunosorbent assay (Shanghai Enzyme-Linked Biotechnology Co., Ltd.) according to the protocols of the manufacturer. Crude enzyme extractions containing frozen tissue powder (0.5 g) were homogenized in 4.5 ml of 0.01 M PBS (pH = 7.2) extracting solution for CCoAOMT, COMT, F5H, and CCR. The reaction system included 40 μl sample dilution and 10 μl crude enzymes were incubated for 30 min at 37°C, rinsed five times with washing liquid, and dried. Afterward, 50 μl of enzyme standard reagent was added, incubated for 30 min at 37°C, and rinsed five times again. Then, 50 μl chromogenic agent A and 50 μl chromogenic agent B were added, mixed, and incubated in the dark for 10 min at 25°C. Finally, 50 μl of termination solution was added. Absorbance was measured at 450 nm, and the amounts of CCoAOMT, COMT, F5H, and CCR were expressed as U mg^–1^ protein.

The activity of cinnamyl-alcohol dehydrogenase (CAD) was determined following the method of Goffner et al. (1992) with appropriate modifications. Frozen tissue powder (2 g) was placed in 2 ml of 0.2 M Tris–HCl (pH = 7.5) extracting solution for CAD crude enzyme extraction. The reaction system included 1 ml of 2 mM NADP, 1.4 ml of 1 mM *trans-*cinnamic acid, and 600 μl of crude enzyme. The mixture was incubated for 30 min at 37°C, and then the reaction was terminated by adding 200 μl of 1 M HCl. Absorbance was measured at 340 nm and the activity of CAD was expressed as U mg^–1^ protein.

### Protein Content Assay

The protein content was determined following the method of Bradford (1976) using bovine serum albumin as standard.

### Quantification of Phenolic Acid and Lignin Monomer Content

Phenolic acid and lignin monomer contents were determined using the method of Fuad and Imad (2015) with appropriate modifications. Frozen tissue powder (3 g) was homogenized in 2 ml of methanol (75%, v/v). Ultrasonic extraction was carried out for 1 h at ambient temperature (22°C ± 2°C; RH, 55–60%). The homogenates were centrifuged at 10,000 × *g* for 40 min, and 3 ml of the supernatant was evaporated for 5 h at 40°C in a low-temperature rotary evaporator. The concentrate was redissolved in 1 ml of mobile phase and filtered through a 0.22-μm microporous membrane for HPLC analysis. A Waters Symmetry^®^ C18 (4.6 × 250 mm, 5 μm) column (Ultra-fast Liquid chromatography from ACQUITY Arc, Waters, United States) was used with a quadruple gradient. The elution velocity was 0.8 ml min^–1^ and the injection volume was 5 μl. Sinapic acid and caffeic acid were assayed at 325 nm. Ferulic acid, *p*-coumaric acid, cinnamic acid, *p-*coumaryl alcohol, sinapyl alcohol, and coniferyl alcohol were assayed at 322, 310, 276, 322, 273, and 263 nm, respectively. The content of phenolic acids and *p-*coumaryl alcohol, sinapyl alcohol, and coniferyl alcohol was calculated using peak time and peak area of the mixture of sample as a standard curve. Results are represented on a fresh weight basis, and the monomer content was expressed as mg kg^–1^.

### Analysis of Total Phenolic Compounds and Lignin Contents

The amount of total phenolic compounds was assayed using the method of Chen et al. (2015) with appropriate modifications. Frozen tissue powder (1 g) was placed in 10 ml of 0.5% acetic acid 70% acetone extracting solution and incubated in the dark for 24 h. The homogenates were centrifuged at 10,000 × *g* for 40 min at 4°C. The reaction system included 1 ml of extracting solution, 2 ml of Folin–Ciocalteu reagent (1:10 dilution), and 2 ml of 7.5% (w/v) sodium carbonate. It was incubated for 5 min at 50°C with methanol as control. Absorbance was measured at 760 nm, and the content of total phenolic compounds was evaluated using a gallic acid standard curve. Results are represented on a fresh weight basis, and the total phenolic compound content was expressed as g kg^–1^.

The amount of lignin was measured following the method of Morrisson (1972) with appropriate modification. Frozen tissue powder (3 g) was placed in 5 ml of 95% ethanol and centrifuged at 10,000 × *g* for 10 min at 4°C. The sediment was rinsed thrice with 95% ethanol and thrice with ethanol:normal hexane = 1:2 (v/v) and then dried. The reaction system included the precipitate and 1 ml of 25% acetyl bromide glacial acetic acid solution, and it was incubated in a 70°C water bath for 30 min. The reaction was stopped by adding 1 ml of 2 M NaOH, after which 2 ml of glacial acetic acid and 200 μl of 7.5 M hydroxylamine hydrochloric acid were added. The homogenates were centrifuged at 10,000 × *g* for 25 min at 4°C. Glacial acetic acid was added to 20 μl of supernatant to bring the total volume to 5 ml. Absorbance was assayed at 280 nm. Lignin content was represented as OD_280_ kg^–1^.

### Statistical Analysis

All experiments were performed at least thrice. Results were represented as mean ± standard error (Origin 8.5). The difference between treatments was analyzed using ANOVA and SPSS 18.0 (SPSS Inc., Chicago, IL, United States) for Duncan’s multiple range test at the 5% level.

## Results

### Effect of NaHS on *P. expansum* Colonization and Increased Endogenous H_2_S, NO, and H_2_O_2_ Content

**FIGURE 1:**
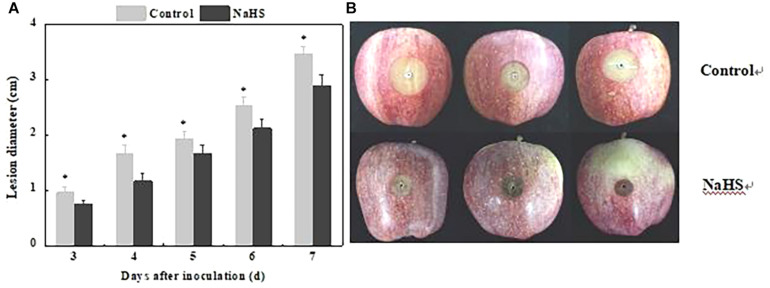
Inhibition of *Penicillium expansum*-induced decay in apples by NaHS. The bars represent the standard error of the mean (±SE). The lesion diameter **(A)** and representative photographs were taken after 5 days **(B)**. ^∗^Significant differences at *P* < 0.05.

The lesion diameter is an important indicator reflecting resistance of fruit. NaHS treatment reduced lesion diameter of apple fruit, which decreased by 32.25% compared with the control at 4 days of treatment (*P* < 0.05) (Figure 1).

**FIGURE 2:**
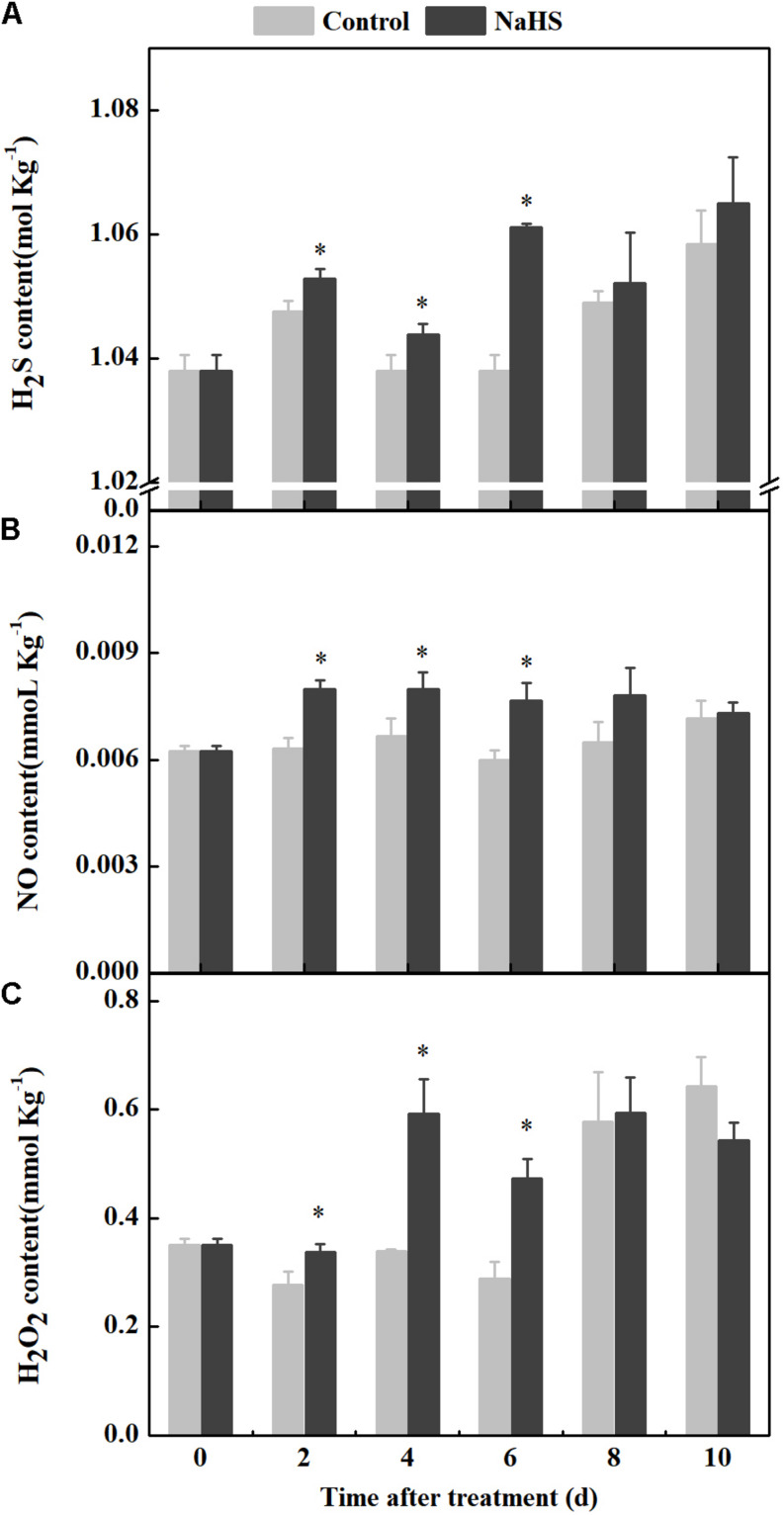
Changes in endogenous H_2_S, NO, and H_2_O_2_ contents in apples after NaHS treatment. H_2_S **(A)**, NO **(B)**, and H_2_O_2_ **(C)**. The light gray and black bars represent the control and NaHS treatments, respectively. The bars represent the standard error of the mean (± SE). ^∗^Significant differences at *P* < 0.05.

NaHS treatment enhanced the endogenous H_2_S level in fruit after 2 days of treatment (Figure 2A). Moreover, the level of endogenous NO in the NaHS-treated fruit increased after 2 days of treatment, which was 26.39 and 27.86% higher than that in the control at 2 and 6 days of treatment, respectively (Figure 2B). The H_2_O_2_ content increased in the NaHS-treated fruit after 2 days of treatment and peaked at 4 days of treatment, which was 74.29% higher than that of the control. At 6 days of treatment, compared with the control, the H_2_O_2_ content increased by 64.08% in the treated fruit (Figure 2C). Taken together, the results indicate that NaHS treatment of apples facilitated the generation of endogenous H_2_S, NO, and H_2_O_2_.

### NaHS Treatment Activated the Phenylpropanoid Pathway and Phenolic Compound Accumulation

**FIGURE 3:**
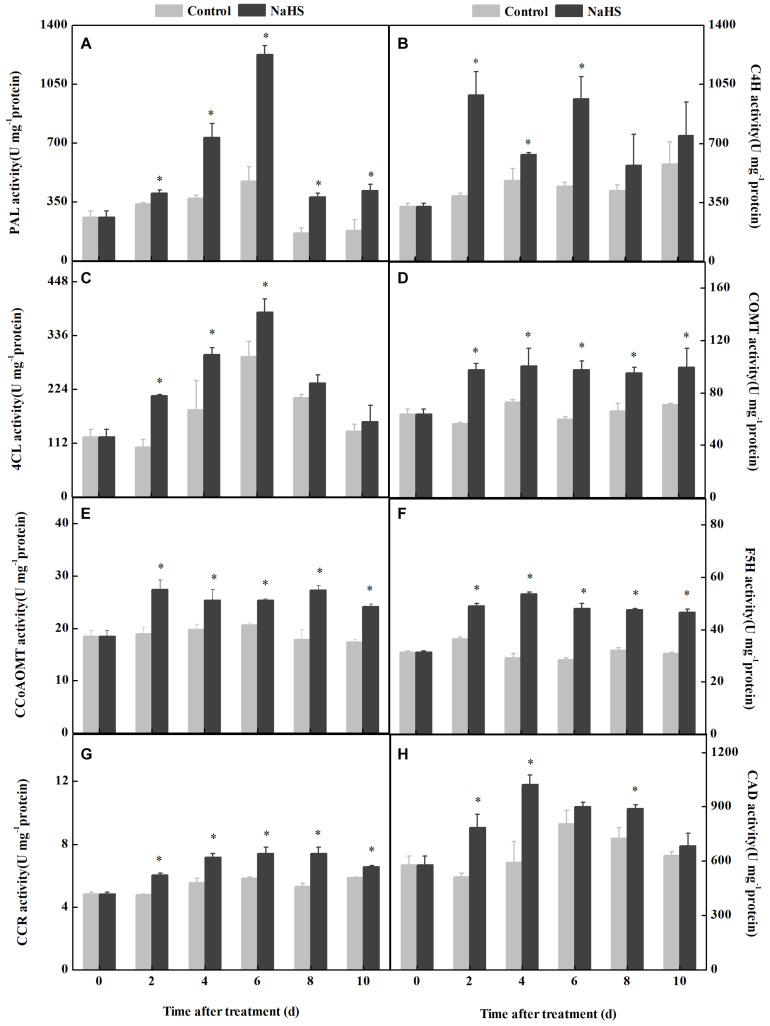
Changes in the activity of the key enzyme of the phenylpropanoid pathway in apples after NaHS treatment. PAL **(A)**, C4H **(B)**, 4CL **(C)**, COMT **(D)**, CCoAOMT **(E)**, F5H **(F)**, CCR **(G)**, and CAD **(H)**. The light gray and black bars represent the control and NaHS treatments, respectively. The bars represent the standard error of the mean (± SE). ^∗^Significant differences at *P* < 0.05.

The activities of key enzymes involved in phenylpropanoid metabolism are critical for regulating fungal colonization (Figure 3). NaHS treatment enhanced the activities of PAL, C4H, 4CL, COMT, F5H, CCoAOMT, CCR, and CAD in apples compared with the control. Among them, the maximum change was found in the activity of PAL, which was 158% higher in the NaHS-treated fruit compared with the control at 6 days of treatment (Figure 3A), while the minimum change was 4CL activity, which was 31.0% higher in the treated fruit compared with the control at 4 days of treatment(Figure 3C).

**FIGURE 4:**
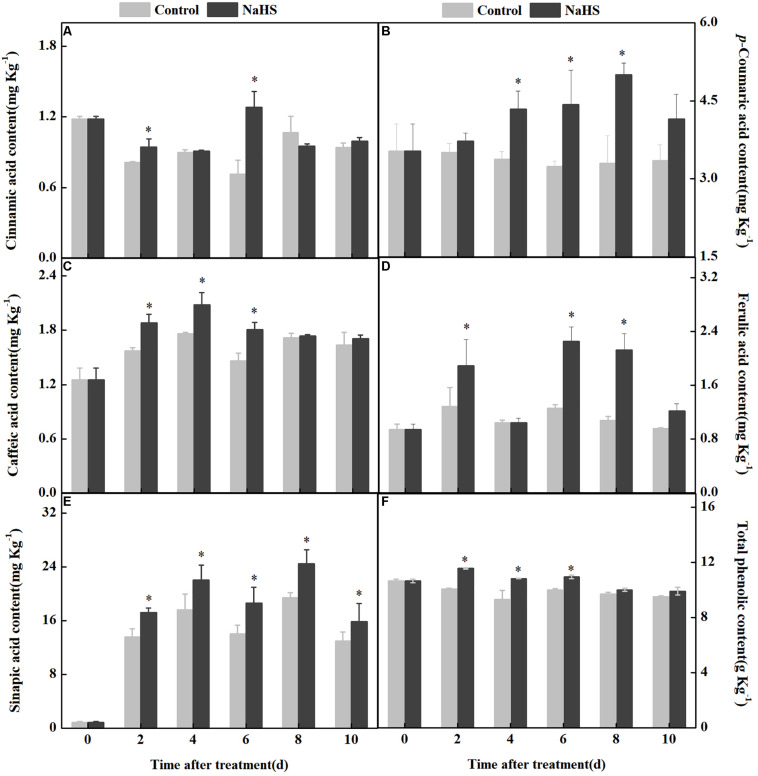
Changes in the content of five phenolic acids and total phenolic compounds in apples after NaHS treatment. Cinnamic acid **(A)**, *p*-coumaric acid **(B)**, caffeic acid **(C)**, ferulic acid **(D)**, sinapic acid **(E)**, and total phenolic compounds **(F)**. The light gray and black bars represent the control and NaHS treatments, respectively. The bars represent the standard error of the mean (± SE). ^∗^Significant differences at *P* < 0.05.

Phenolic acids are mainly generated by the phenylpropanoid pathway. The contents of *p*-coumaric, sinapic, ferulic, and caffeic acid in fruits were increased by NaHS treatment after 2 days of treatment. The content of *p*-coumaric acid in the NaHS-treated fruit was 51.0% higher than that in the control at 8 days of treatment. Compared with the control, the range of changes in the sinapic, ferulic, and caffeic acid content of the treated fruit was around 15% during storage. In addition, the cinnamic acid content fluctuated in the two groups during storage. The total content of phenolic compounds in the treated fruits increased after 2 days of treatment compared with the control (Figure 4F). These results indicated that NaHS treatment affected phenylpropanoid pathway by altering the contents of five phenolic acids and the total phenolic compound content.

### NaHS Treatment Promoted the Synthesis of Lignin Monomers and Lignin

**FIGURE 5:**
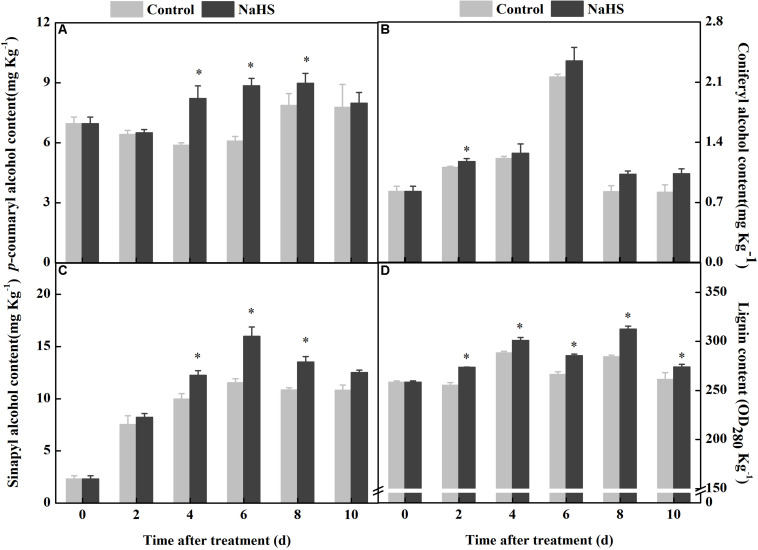
Changes in the content of *p-*coumaryl alcohol, coniferyl alcohol, sinapyl alcohol, and lignin in apples after NaHS treatment. *p-*Coumaryl alcohol **(A)**, coniferyl alcohol **(B)**, sinapyl alcohol **(C)**, and lignin **(D)**. The light gray and black bars represent the control and NaHS treatments, respectively. The bars represent the standard error of the mean (± SE). ^∗^Significant differences at *P* < 0.05.

Lignin is composed of *p*-coumaryl, sinapyl, and coniferyl alcohols, which all belonged to monolignols. The contents of *p*-coumaryl, sinapyl, and coniferyl alcohols in the fruit increased and consisted of the process of wound healing, while the NaHS treatment enhanced the accumulation of these compounds. At 6 days of treatment, the contents of *p*-coumaryl and sinapyl alcohols increased by 45 and 39% compared with the control, respectively (Figures 5A,C). At 8 days of treatment, the content of coniferyl alcohol in the NaHS-treated fruit was 25% higher than that in the control (Figure 5B). Moreover, the peak of lignin content was found at 4 days of treatment in the treated fruit, which was earlier and higher compared with the control (Figure 5D). These results indicated that the NaHS treatment enhanced the accumulation of *p-*coumaryl alcohol, sinapyl alcohol, coniferyl alcohol, and lignin in fruits.

## Discussion

In this study, we have described a new mechanism by which NaHS treatment promotes the generation of endogenous H_2_S, NO, and H_2_O_2_ in apples, thus modulating the mechanism of host resistance (Figure 2). These results are similar to the observations that NaHS increases the contents of endogenous H_2_O_2_ and NO in wheat (Karpets et al., 2020) and H_2_S and NO in tomato seedlings (Liang et al., 2018). NaHS is used as a postharvest treatment to delay senescence in kiwifruits (Zhu et al., 2014), mulberries (Hu H. et al., 2014), and strawberries (Hu et al., 2012). At 1 ppm, H_2_S has no negative effects on human health, and the enzymes in the human body can detoxify H_2_S by oxidizing it to harmless sulfate (Wang, 2012).

NaHS is an exogenous H_2_S donor that dissociates into Na^+^ and HS^–^ when dissolved in water; HS^–^ then combines with H^+^ to generate H_2_S (Hosoki et al., 1997). L-cysteine and D-cysteine desulfhydrase are important enzymes for endogenous H_2_S generation and are activated by exogenous H_2_S in plants (Hu H. et al., 2014). L/D-CD can degrade cysteine into H_2_S, pyruvate, and ammonium (Wang, 2012). Endogenous H_2_S activates nitrate reductase (NR), which turns NAD(P)H and nitrate into NAD^+^ and H_2_O. Nitrite is unstable and rapidly decomposes to form NO (Besson-Bard et al., 2008; Liang et al., 2018). In addition, H_2_S can activate NADPH oxidase (NOX), which forms O_2_^–^ by transferring electrons, which then produces H_2_O_2_ *via* superoxide dismutase (SOD) (Karpets et al., 2020). Therefore, we infer that NaHS promotes endogenous H_2_S production by activating L/D-CD activity. H_2_S accelerates the formation of H_2_O_2_ by activating NOX and SOD, resulting in induced resistance. As a signal molecule, H_2_O_2_ activates NR and facilitates the synthesis of endogenous NO. The potential mechanism of NaHS in promoting the production of endogenous H_2_S, NO, and H_2_O_2_ remains to be identified.

The possibility that NaHS modulates H_2_O_2_ raised the question of whether it will also modulate the phenylpropanoid pathway in apples. Phenylpropanoid metabolism, which generates phenolic acids and monolignols, plays a vital role in inducing stress resistance against biotic factors. Phenolic acids have shown antifungal and antioxidant activity (Romanazzi et al., 2016). PAL is a crucial enzyme in the phenylpropanoid metabolism, catalyzing the conversion of L-phenylalanine into cinnamic acid (Deng and Lu, 2017). C4H catalyzes the synthesis of *p*-coumaric acid from cinnamic acid and caffeic acid from *p*-coumaric acid. Sinapic acid is produced from caffeic acid by COMT and F5H (Arrieta-Baez and Stark, 2006; Vogt, 2010). In this study, we identified that NaHS treatment markedly enhanced the activities of PAL, C4H, 4CL, COMT, and F5H (Figures 3A–D,F) and accelerated the synthesis of cinnamic, *p*-coumaric, ferulic acid, and total phenolic compounds in apples (Figure 4). These results were similar to those where H_2_S treatment in *Linum album* roots increases PAL activity and accelerates the accumulation of ferulic acid and total phenolic compounds (Fakhari et al., 2019). Given that H_2_S promoted the synthesis of NO and H_2_O_2_, it is understandable that it also activated the phenylpropanoid pathway (Fakhari et al., 2019). A previous study showed that the gene expression and activity of PAL, C4H, and 4CL were upregulated by NO and that it elevated the generation of total phenolic and lignin in peach (Li G. J. et al., 2016). In addition, H_2_O_2_ activates PAL, C4H, and 4CL; promotes the synthesis of lignin; and accelerates the lignification process in bamboo shoot (Li D. et al., 2019). Therefore, we infer that H_2_S, NO, and H_2_O_2_ could serve as signal molecules to activate the phenylpropanoid pathway in the apple system.

*p*-Coumaric acid is a significant bridge connecting the phenylpropanoid and lignin pathways. Lignin is primarily composed of coniferyl, *p*-coumaroyl, and sinapyl alcohol, which are generated from *p*-coumaroyl-CoA, feruloyl-CoA, and sinapaldehyde, respectively, by an enzyme cascade of CCR and CAD or CCR (Vanholme et al., 2010). They undergo oxidative crosslinking to form guaiacyl lignin, syringyl lignin, and parahydroxyphenyl lignin, respectively (Zhao and Dixon, 2011; Chezem and Clay, 2016). The present study indicated that NaHS treatment improves the activities of CCoAOMT, F5H, COMT, CCR, and CAD (Figures 3D–H) and promotes the accumulation of *p*-coumaryl, coniferyl, sinapyl alcohols, and lignin in apples (Figure 5). It has been reported that NO not only activates PAL, C4H, and 4CL but also increases the content of lignin in muskmelon (Yan et al., 2019). In addition, H_2_O_2_ improves the synthesis of lignin in bamboo shoots (Li D. et al., 2019). Therefore, we speculate that H_2_S promotes the synthesis of phenolic acids, increases enzyme activity in lignin synthesis, and accelerates the accumulation of lignin and monolignols that could contribute to the enhanced resistance against the pathogen *P. expansum*.

## Conclusion

*Penicillium expansum* is a wound pathogen, and enhanced phenylpropanoid metabolic activation in apples may contribute to the prevention of *P. expansum* colonization. NaHS treatment enabled the generation of endogenous H_2_S, NO, and H_2_O_2_ in apples. Moreover, NaHS supported the activities of PAL, C4H, 4CL, COMT, F5H, CCoAOMT, CCR, and CAD and induced the accumulation of cinnamic, *p*-coumaric, caffeic, ferulic, and sinapic acids and total phenolic compounds. NaHS treatment also accelerated the accumulation of *p*-coumaryl, coniferyl, and sinapyl alcohols and lignin. Given that NaHS fumigation on apples is safe for human consumption, this treatment could be considered as a possible postharvest treatment to induce resistance against *P. expansum* in apples.

## Data Availability Statement

The raw data supporting the conclusions of this article will be made available by the authors, without undue reservation.

## Author Contributions

HD wrote the manuscript. YB reviewed and edited the manuscript and was the project administrator and supervisor. BW carried out data curation and investigation. YL analyzed the data. LM provided experiment assistance. YZ and DP edited and organized the manuscript. All authors contributed to the article and approved the submitted version.

## Conflict of Interest

The authors declare that the research was conducted in the absence of any commercial or financial relationships that could be construed as a potential conflict of interest.

## Publisher’s Note

All claims expressed in this article are solely those of the authors and do not necessarily represent those of their affiliated organizations, or those of the publisher, the editors and the reviewers. Any product that may be evaluated in this article, or claim that may be made by its manufacturer, is not guaranteed or endorsed by the publisher.
